# Attenuation of Renovascular Damage in Zucker Diabetic Fatty Rat by NWT-03, an Egg Protein Hydrolysate with ACE- and DPP4-Inhibitory Activity

**DOI:** 10.1371/journal.pone.0046781

**Published:** 2012-10-10

**Authors:** Yumei Wang, Sjoerd Landheer, Wiek H. van Gilst, Aart van Amerongen, Hans-Peter Hammes, Robert H. Henning, Leo E. Deelman, Hendrik Buikema

**Affiliations:** 1 5th Medical Department, Section of Endocrinology, University Hospital Mannheim, University of Heidelberg, Mannheim, Germany; 2 Departments of Clinical Pharmacology, University Medical Center Groningen, University of Groningen, Groningen, The Netherlands; 3 Department of Cardiology, University Medical Center Groningen, University of Groningen, Groningen, The Netherlands; 4 Food and Biobased Research, Wageningen University and Research Center, Wageningen, The Netherlands; Paris Institute of Technology for Life, Food and Environmental Sciences, France

## Abstract

**Background:**

Dipeptidyl peptidase 4 (DPP4) and angiotensin-converting enzyme (ACE) are important target enzymes in glycemic control and renovascular protection. Here, we studied the effect of NWT-03, an egg protein hydrolysate with DPP4- and ACE-inhibitory activity, on renovascular damage in Zucker diabetic fatty (ZDF) rats. Comparisons were made to rats treated with vildagliptin (VIL), included as a positive control for the effect of DPP4 inhibition.

**Methods:**

ZDF rats received NWT-03 (1 g/kg/day) or VIL (3 mg/kg/day) from 10 to 25 weeks of age. Metabolic and renal functions were assessed; the kidney was removed for histological analysis of glomerulosclerosis and expression of pro-inflammatory/fibrotic markers (RT-PCR and Western blotting); and the aorta was removed for studies of endothelium-dependent relaxation (EDR).

**Findings:**

Hyperinsulinemic ZDF rats typically developed signs of type-2 diabetes and renovascular damage, as evidenced by albuminuria, glomerulosclerosis, and impaired EDR. Neither NWT-03 nor VIL improved metabolic parameters; for VIL, this was despite a 5-fold increase in glucagon-like peptide (GLP)-1 levels. NWT-03 and VIL both reduced renal interleukin (Il)-1β/Il-13 mRNA expression and glomerulosclerosis. However, only NWT-03 additionally decreased renal tumor necrosis factor (TNF)-α mRNA and P22^phox^ protein expression, reduced albuminuria, and restored aortic EDR. Indomethacin added to the organ bath instantly improved aortic EDR, indicating a role for cyclooxygenase (COX)-derived contractile prostanoids in opposing relaxation in ZDF rats. This indomethacin effect was reduced by NWT-03, but not by VIL, and coincided with decreased renal COX-1/2 protein expression.

**Conclusion and Interpretation:**

Long-term supplementation with the egg protein hydrolysate NWT-03 attenuated renovascular damage in this preclinical rat model of type 2 diabetes. A comparison to the DPP4-inhibitor VIL suggests that the effects of NWT-03 were related to both ACE- and DPP4-inhibitory properties. The development of protein hydrolysates with a multiple-targeting strategy may be of benefit to functional food formulations.

## Introduction

Food-derived bioactive peptides represent a source of health-enhancing components that may be incorporated in functional foods. The intrinsic bioactivities of the peptides encrypted in major food proteins are latent until released and activated by enzymatic hydrolysis, for example during gastrointestinal digestion [1]. Quantitative *in silico* analyses may be used to calculate bioactivity in protein digests and identify good potential sources of peptides of interest [2], [3]. In such a search, we identified the egg protein lysozyme as a potential precursor protein of angiotensin-converting enzyme (ACE)-inhibitory peptides upon digestion with alcalase. The activity of this hydrolysate, termed NWT-03, was confirmed in subsequent *in vitro* ACE-inhibition assays (IC_50_ = 0.07 mg/mL) (Buikema et al., unpublished data). ACE-inhibitors have emerged as important agents, not only in the management of hypertension but also for their potential to reduce cardiovascular risk and nephropathy; moreover, they provide renovascular protection through different mechanisms beyond their primary therapeutic actions [4], [5]. This appears to be of particular importance in patients with diabetes in whom kidney disease is one of the most serious complications.

Interestingly, our *in vitro* assays additionally highlighted the potential of NWT-03 to inhibit dipeptidyl peptidase 4 (DPP4) activity (IC_50_ = 0.9 mg/mL) (Buikema *et al.*, unpublished data). The latter enzyme degrades active glucagon-like peptide 1 (GLP-1), a pro-glucagon-derived hormone secreted by intestinal L-cells and pancreatic α-cells, that has profound effects on gut clearing and insulin secretion in the gastrointestinal tract [6]. Because of this action, stable GLP-1 analogues (e.g., exenatide) and DPP4 inhibitors, such as vildagliptin (VIL), have been developed for the treatment of type 2 diabetes mellitus (DM2) [7]. Previous studies with insulin-resistant rats have suggested that chronic treatment with synthetic DPP4-inhibitors may improve impaired glucose metabolism and dyslipidemia [8].

Whereas research in the field of bioactive peptide analysis and functional food development has mostly followed single-target strategies, network models suggest that partial inhibition of a small number of targets can be more efficient than the complete inhibition of a single target [9]. Given this, we hypothesized that the egg protein hydrolysate NWT-03, which displays both ACE- and DPP-4 inhibitory activity, might beneficially affect parameters of renovascular damage development in DM2. To study this, we supplemented the drinking water of Zucker diabetic fatty (ZDF) rats with NWT-03 for 15 weeks, and then assessed renal damage and vascular endothelial dysfunction. A parallel group of ZDF rats treated with the commercially available DPP4-inhibitor VIL, included as a positive control for the effect of DPP4-inhibition, was studied concurrently.

## Materials and Methods

### Animals and study design

The protocols for animal care and use were in accordance with the NIH Guide for the Care and Use of Laboratory Animals and were approved by the Committee for Animal Experiments (Dierexperimentencommissie, DEC) of the University of Groningen (Permit Number: DEC4809A and −5370A). Studies were conducted using male obese ZDF (*fa/fa*) and Zucker lean control (+/?) rats obtained from Charles River (France). Animals were housed group-wise in standard cages and maintained on a 12∶12-hour light∶dark cycle with free access to food (Purina 5008) and water. After acclimatization, 10-week-old ZDF rats were randomly assigned to receive 1 g/kg per day NWT-03 (n = 9) or 3 mg/kg per day VIL (n = 7), or remained untreated (ZDF; n = 7). In preliminary *in vivo* studies, oral administration of NWT-03 and VIL as a single dose at the indicated concentrations induced a significant inhibition of plasma DPP4-activity, an effect that was more pronounced after VIL (Figure S1). Furthermore, in spontaneously hypertensive rats (SHRs), 1 g/kg NWT-03 was comparably effective in inhibiting plasma ACE-activity and reducing systolic blood pressure after acute oral gavage (Figure S2).

Long-term supplementation with NWT-03 or VIL via drinking water was maintained for 15 weeks, during which body weight and water intake were monitored and the amount of NWT-03 and VIL were adjusted accordingly. Samples for determining metabolic parameters were collected at the start and the end of the study period. For the collection of urine, rats were housed individually in a metabolic cage for 24 hours, and samples were analyzed for glucose, albumin, and malondialdehyde (MDA). Blood samples, collected from the tail vein during mild anesthesia (2.0–2.5% isoflurane in oxygen), were analyzed for blood glucose, HbA1c, cholesterol, free fatty acids (FFAs), and serum insulin; in addition, GLP-1 levels were determined at the end of the study period. Animals were sacrificed at the age of 25 weeks under anesthesia, as described above. Systolic blood pressure and heart rate were measured using a catheter-tip micro-manometer (Millar Instruments, Germany) advanced into the aorta. A final blood sample was drawn and the kidneys were flushed with saline, after which kidneys and aorta were removed together. Part of the kidney was fixed with 4% formaldehyde and another part was snap-frozen in liquid nitrogen and stored at −80°C. The aorta was studied for endothelium-dependent relaxation (EDR) responses in organ bath studies.

### Bioanalytical procedures

Blood glucose and cholesterol were determined using Accu-Chek Aviva and AccuTrend (both Roche Diagnostics, Almere, The Netherlands) monitors, respectively; Hb1Ac was determined using a DCA Vantage Analyzer (Siemens Healthcare Diagnostics Inc., Deerfield, IL). Serum GLP-1 (Glucagon-Like Peptide-1 [Active] ELISA Kit; Millipore Corporation, Billerica, MA), insulin ([Rat] enzymatic immunoassay; ALPCO Diagnostics, Salem, NH), and FFAs (Free Fatty Acid Quantification Kit; Biovision, Research Products, Mountain View, CA) were determined using the indicated commercial kits. Urinary glucose was confirmed using a Diabur 5000 dipstick (Roche Diagnostics); color intensity was categorized from 0 to 7, covering a range of 0–5% (g/dL) glucose. Urinary albumin and creatinine were determined using a DCA Vantage Analyzer (Siemens Healthcare Diagnostics Inc.), and urinary MDA levels were assessed using a Malondialdehyde Assay kit (NWK-MDA01; Northwest Life Science Specialties, LLC, Vancouver, WA).

### Light microscopy

Coronal tissue slices through the mid portion of the kidney were fixed in 4% formaldehyde and embedded in paraffin, after which sections (4 µm) were stained with periodic acid-Schiff (PAS) [10]. Subsequently, glomeruli were semi-quantitatively scored for focal glomerulosclerosis (FGS) by light microscopy on a scale of 1 to 4, as described previously [11]. FGS was scored positive when mesangial expansion, mesangial cellularity, adhesion formation, and capillary obliteration was present in one segment. If 25% of glomerulus was affected, a score of 1 was adjudged, 50% was scored as 2, 75% as 3, and 100% as 4. The ultimate score is then obtained by multiplying the degree of change by the percentage of glomeruli with the same degree of injury and adding these scores. A total of 50 glomeruli per kidney were scored moving from cortex to medulla and the average value per kidney (i.e. per animal) calculated.

### Immunohistochemistry

Immunohistochemistry was performed on paraffin-embedded sections, as described previously [12]. After the section was deparaffinized and dehydrated, two-step immunoperoxidase staining was performed according to standard methods. Peroxidase activity was developed by incubation with 3-amino-9-ethylcarbazole (AEC; Dakopatts, Glostrup, Denmark). Negative controls were performed by replacing primary antibodies with PBS. The antibodies used were mouse monoclonal anti-α-smooth muscle actin (α-SMA; DAKO, Denmark) for myofibroblasts, and rabbit polyclonal anti-KIM-1 (kidney injury molecule 1; generously provided by Dr. H. van Goor, University Medical Center Groningen, The Netherlands) for proximal tubular damage/interstitial damage. To evaluate pre-fibrosis after diabetic injury, the expression of α-SMA was measured using computer-assisted morphometry. Total staining was evaluated at a magnification of 400× using 15–20 fields per section. Glomeruli and arteries were excluded from measurements. α-SMA staining was divided by the area measured and expressed as a percentage, and an average score calculated per section. The expression of KIM-1, a marker of tubular damage, was also measured using computer-assisted morphometry. Total staining was evaluated at a magnification of 200× using 25 fields per section and expressed as average intensity of staining. Evaluation of the stainings and morphometric analysis were performed in a blinded manner.

### RNA isolation and real-time PCR

Frozen kidney samples containing both cortex and medulla were homogenized, and RNA was isolated using a kit (Qiagen, Venlo, The Netherlands). cDNA was synthesized from total RNA (1 µg) by reverse transcription (RT) and was used to analyze the expression of interleukin (Il)-1β, Il-13, E-selectin, vascular cell adhesion molecule (VCAM)-1, CD68, and tumor necrosis factor (TNF)-α using a real-time polymerase chain reaction (PCR) protocol, as described previously [13]. Quantification was performed using Absolute QPCR SYBR Green reagents (Molecular Probes, Leiden, The Netherlands) and a CFX384 Real-Time PCR Detection System (Bio-Rad Laboratories, Inc., Hercules, CA). Thermal cycling was carried out at 95°C for 15 minutes, followed by 40 cycles with 95°C for 15 seconds and 60°C for 1 minute. Glyceraldehyde 3-phosphate dehydrogenase (GAPDH) was used as a housekeeping gene. The PCR primers used are presented in Table S1.

### Western blotting

Kidney tissues were homogenized in RIPA lysis buffer (composition: 1% NP40, 0.5% sodium deoxycholate, 0.1% sodium dodecyl sulfate (SDS), 10 mM β-mercapto-ethanol, 10 mg/ml PMSF, 5 µl/ml aprotinin, 100 mM sodium orthovanadat, 5 µl/ml benzamidine, 5 µl/ml pepstatine A, 5 µl/ml leupeptine in 1× PBS) on ice using an Ultra Turrax homogenizer, after which the supernatants were collected and protein concentrations were determined. After separating samples (50 µg) on 4–20% Precise Protein Gels (Pierce, Rockford, IL), proteins were electrotransferred onto nitrocellulose membranes (Bio-Rad), and nonspecific binding was blocked by incubating for 30 minutes with with 5% skimmed milk in PBS. Thereafter, membranes were incubated with primary antibodies overnight at 4°C, and then with secondary antibodies for 1 hour at room temperature. Immunoreactive proteins were visualized by enhanced chemiluminescence (SuperSignal West Pico Chemiluminescent Substrate; Thermo Scientific, Pierce, Rockford, IL). The primary antibodies used were rabbit polyclonal anti-P22*^phox^* (Santa Cruz Biotechnology, Santa Cruz, CA), rabbit polyclonal anti-COX-1 (cyclooxygenase-1; Alexis, San Diego, CA), mouse monoclonal anti-COX-2 (BD Pharmingen, San Diego, CA), rabbit polyclonal anti-TXA_2_R (thromboxane A2 receptor; Santa Cruz Biotechnology), and mouse monoclonal GAPDH (Sigma, St. Louis, MO).

### Vascular studies with isolated aortic rings

For isotonic displacement studies, the aorta was cleaned of fat and connective tissue, cut into rings, and mounted in an organ bath setup filled with Krebs solution bubbled with 95%O_2_/5%CO_2_ at 37°C, as described previously [14]. Briefly, rings were pre-constricted with 1 µmol/L phenylephrine, and EDR responses (percent of pre-constriction) to 0.01–100 µmol/L acetylcholine (ACh) and maximal endothelium-independent relaxation to 10 µmol/L sodium nitroprusside were studied; all concentrations are final concentrations in the bath. The EDR response to ACh was additionally studied in the presence of 10 µmol/L indomethacin, added to inhibit COX-derived prostanoids, as described previously [15].

### Treatment with drugs and other compounds

NWT-03 was produced by Food & Biomade Research (Wageningen University, The Netherlands). Briefly, a solution of 5% lysozyme protein (Belovo) was hydrolyzed for 6 hours with a 2% (w/w) solution of alcalase enzyme (Novozyme) at an optimal pH (pH 8) and temperature (60°C), After hydrolysis, the enzyme was inactivated, the hydrolysate was centrifuged to remove insoluble protein, and the supernatant was freeze dried. VIL (Galvus Novartis) was obtained from the Hospital Pharmacy at the University Medical Center Groningen, The Netherlands. The KIM-1 antibody was generously provided by Dr. H. van Goor (University Medical Center Groningen, Netherlands). All other reagents were of analytical grade and were purchased from commercial suppliers.

### Statistical analysis

Data are expressed as means ± SEMs. Untreated ZDF rats were compared to lean controls using Student's unpaired *t*-test. The three experimental ZDF groups were compared among each other using one-way analysis of variance (ANOVA) in combination with Dunnett *post hoc* analysis for multiple comparisons, using untreated ZDF rats as a control group. Differences with a p-value <0.05 in two-tailed tests were considered significant (SPPS 18.0; SPSS Inc., Chicago, IL).

## Results

### ZDF rats as an animal model of DM2 with renovascular damage (Table 1)

**Table 1:** Characteristics of ZDF and lean control rats at 10 and 25 weeks of age.

	10 weeks		25 weeks	
	Lean (n = 7)	ZDF (n = 7)	Lean (n = 7)	ZDF (n = 7)
Body weight (g)	246±5	342±5***	393±4	427±20
Left kidney weight (mg)	-	-	1263±29	2154±47***
Right kidney weight (mg)	-	-	1335±42	2199±74***
***Hemodynamic parameters***				
Aorta HR (bpm)	-	-	261±9	226±14
Aorta SBP (mmHg)	-	-	117±4	124±4
***Metabolic parameters***				
Glucose (mmol/L)	7.76±0.24	12.33±2.4	9.6±0.42	23.9±1.72***
Hb1Ac (%)	3.51±0.03	5.37±0.25***	3.57±0.04	9.54±0.16***
Insulin (ng/mL)	29.1±2.6	243.3±34.6***	28.3±3.3	28.5±4.0
Cholesterol (mmol/L)	<3.88	4.03±0.08	4.01±0.13	7.33±0.19***
***Urinary parameters***				
Albumin (mg/24 h)	2.8±0.6	33.3±12.1*	1.9±0.5	77.4±15.7**
Glucose (category 0–7)	0.0±0.0	3.0±0.9*	0.0±0.0	>7.0
MDA:Creatinine	3.22±0.27	4.14±0.86	1.74±1.99	4.44±1.03*
24-h Urine volume (mL)	6.7±0.5	20.8±7.1	11.4±1.3	74.8±8.2***

Abbreviations: HR, heart rate; bpm, beats per minute; SBP, systolic blood pressure; MDA, malondialdehyde.

Data are means ± SEMs (*p<0.05, **p<0.01 and ***p<0.001 vs. lean of same age; *t*-test).

#### Metabolic parameters

At 10 weeks of age (the start of the study), ZDF rats already showed increased body weight, first signs of hyperglycemia (elevated blood glucose and HbA1c levels) and hyperlipidemia (FFA: 0.24±0.05 and 0.65±0.18 mmol/L for lean and ZDF, respectively; p<0.05), and increased serum insulin. The additional presence of glucose in the urine (category 3 coloring, corresponding to 0.5% [g/dL]) indicated the onset of diabetes, whereas increased albumin levels in 24-hour urine samples suggested the presence of early renal damage. Metabolic parameters in ZDF rats profoundly worsened over time, with blood cholesterol, glucose, and HbA1c levels doubling after the 15-week study period; the levels of these parameters remained low in lean controls. In contrast, serum insulin declined to lean levels in ZDF rats, indicating progressive insulinopenia. Taken together with a marked increase in urinary volume production and high urinary glucose levels, these observations confirmed the progressive development of a DM2 state in ZDF rats. These changes were accompanied by a significant increase in urinary albumin excretion and higher urinary MDA levels.

#### Renal damage

**Figure 1:**
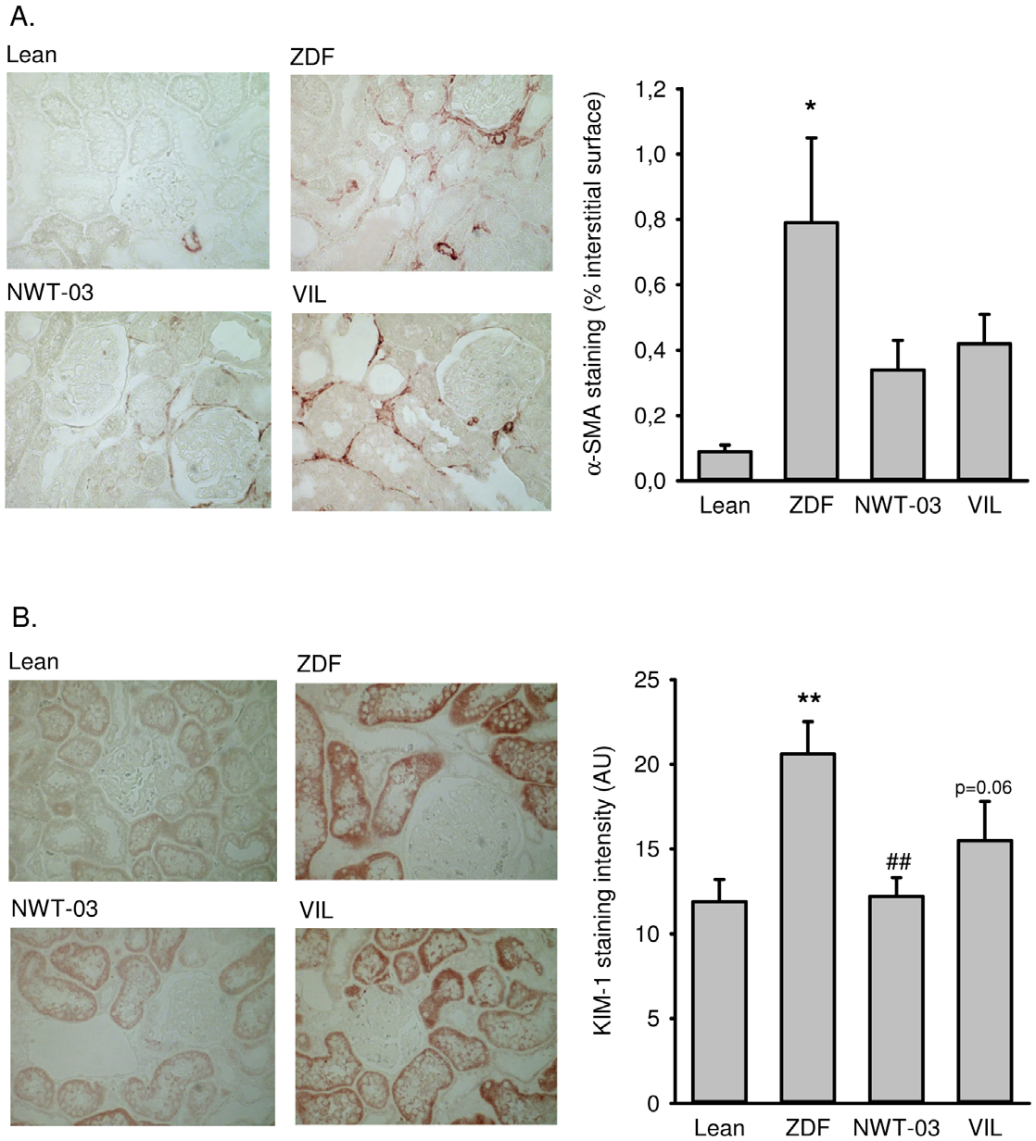
Renal expression of (A) α-SMA and (B) KIM-1. Shown are representative pictures of lean control rats (top left) and untreated ZDF rats (top right), and ZDF rats supplemented with NWT-03 (bottom left) or treated with VIL (bottom right). Bars in the graphs represent the average per group, presented as means ± SEMs (*p<0.05 and **p<0.01 vs lean; ##p<0.01 vs ZDF).

**Figure 2:**
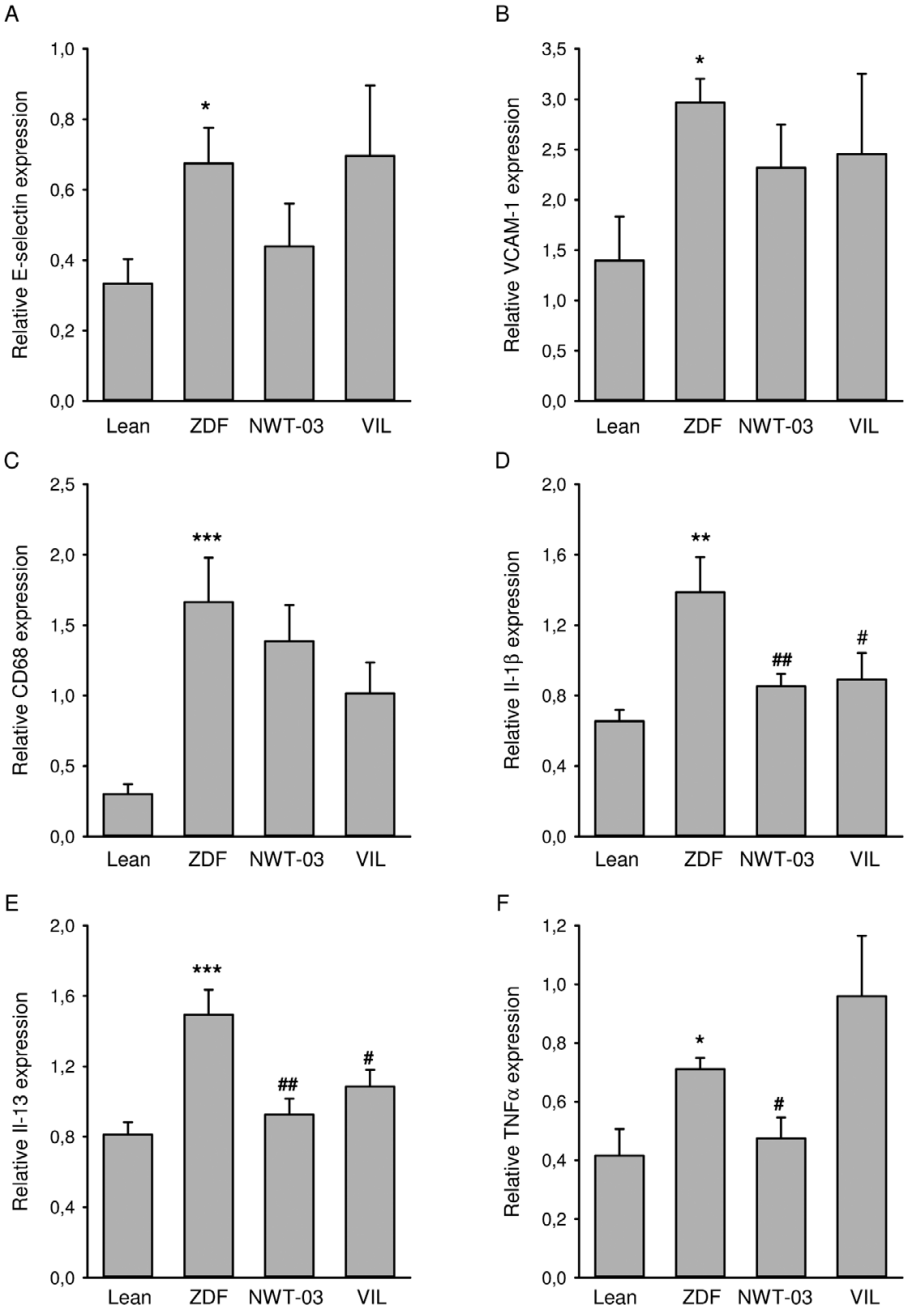
Renal mRNA expression of (A) E-selectin, (B) VCAM-1, (C) CD68, (D) Il-1β, (E) Il-13, and (F) TNFα. Shown are results for lean controls and untreated ZDF rats, and ZDF rats supplemented with NWT-03 or treated with VIL. Target mRNA levels are expressed relative to those of GAPDH and are presented as means ± SEMs (*p<0.05, **p<0.01, and ***p<0.001, vs. lean; #p<0.05 and ##p<0.01 vs. ZDF).

**Figure 3:**
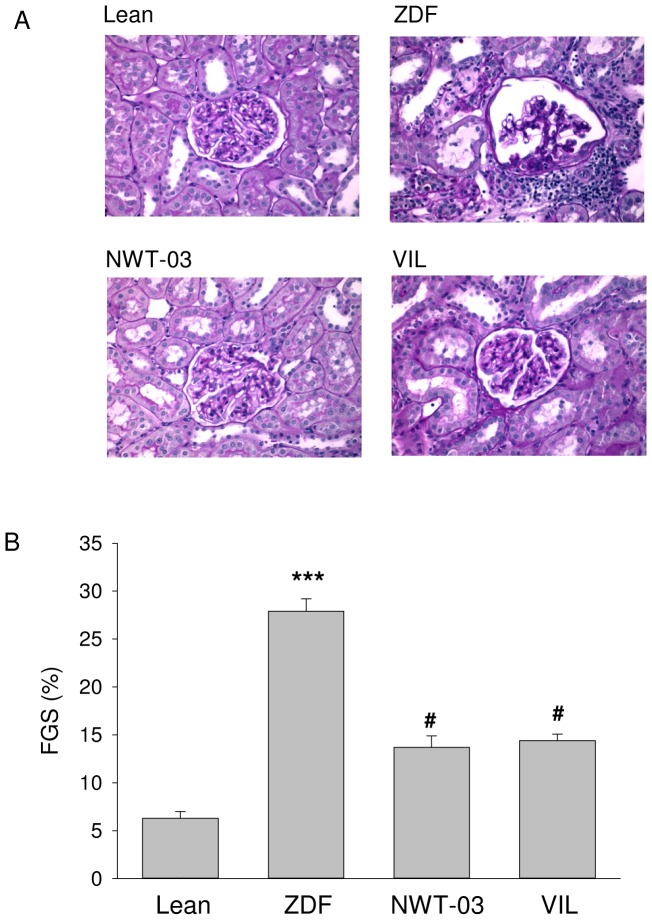
Renal morphology and FGS. (A) Shown are representative sections for lean control rats (top left) and untreated ZDF rats (top right), and ZDF rats supplemented with NWT-03 (bottom left) or treated with VIL (bottom right). (B) FGS (see Methods); data are means ± SEMs (***p<0.001 vs. lean; #p<0.05 vs. ZDF).

**Figure 4:**
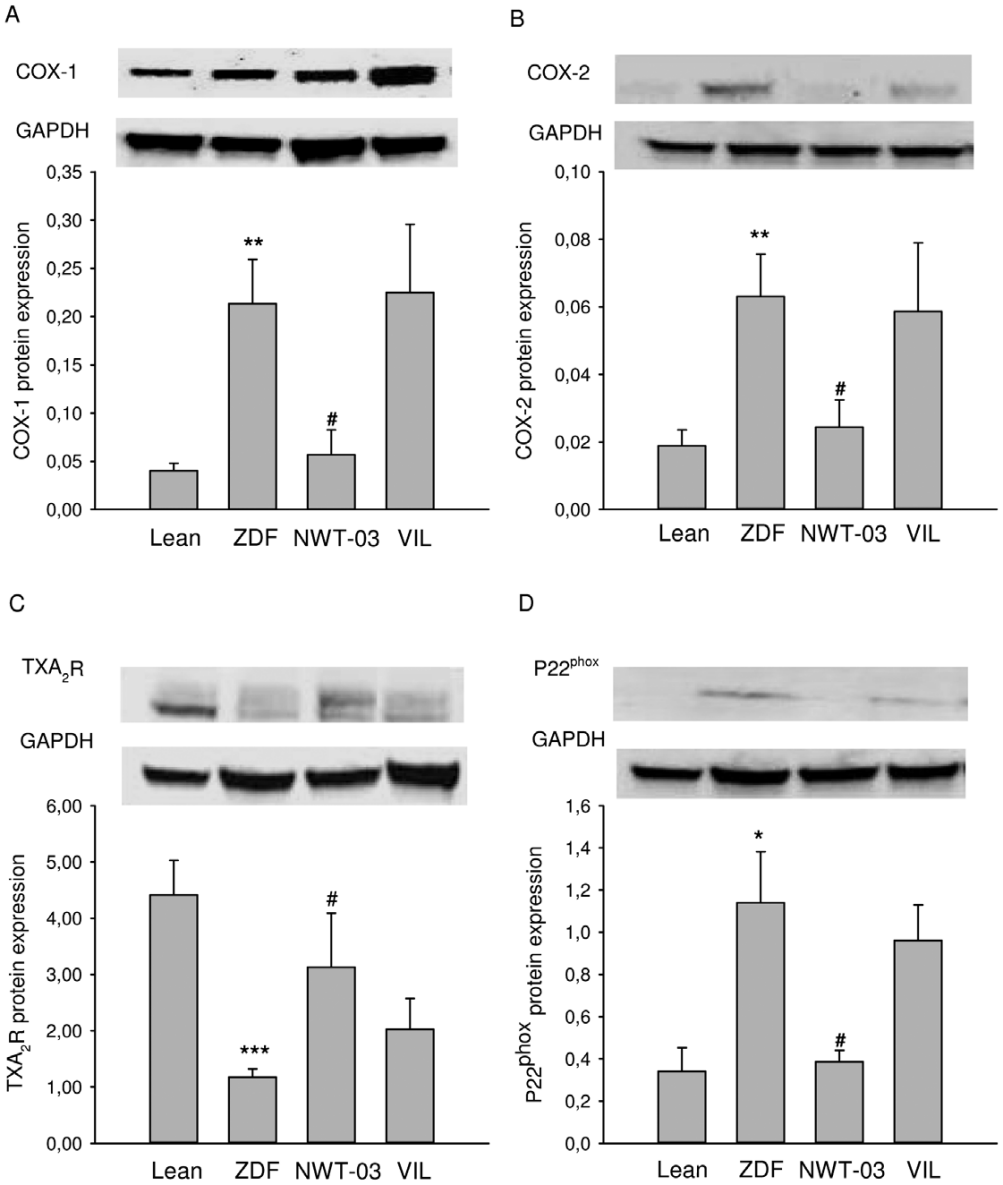
Renal expression of (A) COX-1, (B) COX-2, (C) TXA_2_R, and (D) P22^phox^ protein. Shown are results for lean control and untreated ZDF rats, and ZDF rats supplemented with NWT-03 or treated with VIL. Data are expressed relative to GAPDH and are presented as means ± SEMs (*p<0.05, **p<0.01, and ***p<0.001 vs. lean; #p<0.05 vs. ZDF).

ZDF kidney sections showed positive immunostaining and increased expression of α-SMA and KIM-1 (Figure 1), indicating the presence of mesangial cell proliferation and renal injury, respectively. RT-PCR analyses showed increased renal mRNA expression of all the inflammatory markers studied and the development of structural renal damage. Specifically, compared to lean controls, E-selectin was increased by 2-fold, VCAM-1 by 2.1-fold, CD-68 by 5.5-fold, Il-1β by 2.1-fold, Il-13 by 1.8-fold, and TNFα by 1.7-fold in ZDF rats (Figure 2). Histological analyses of PAS-stained kidney sections showed a 5-fold increase in FGS (Figure 3); kidney weights were also increased in ZDF rats. Western blot analyses additionally revealed a 3.3-fold increase in the abundance of renal P22*^phox^* protein (Figure 4), implicated as a mediator of oxidative stress [16]. Renal COX-1 and COX-2 proteins were also increased (5.3- and 3.4-fold, respectively) in ZDF rats, whereas TXA_2_R was decreased by 3.7-fold (Figure 4).

#### Vascular dysfunction

**Figure 5:**
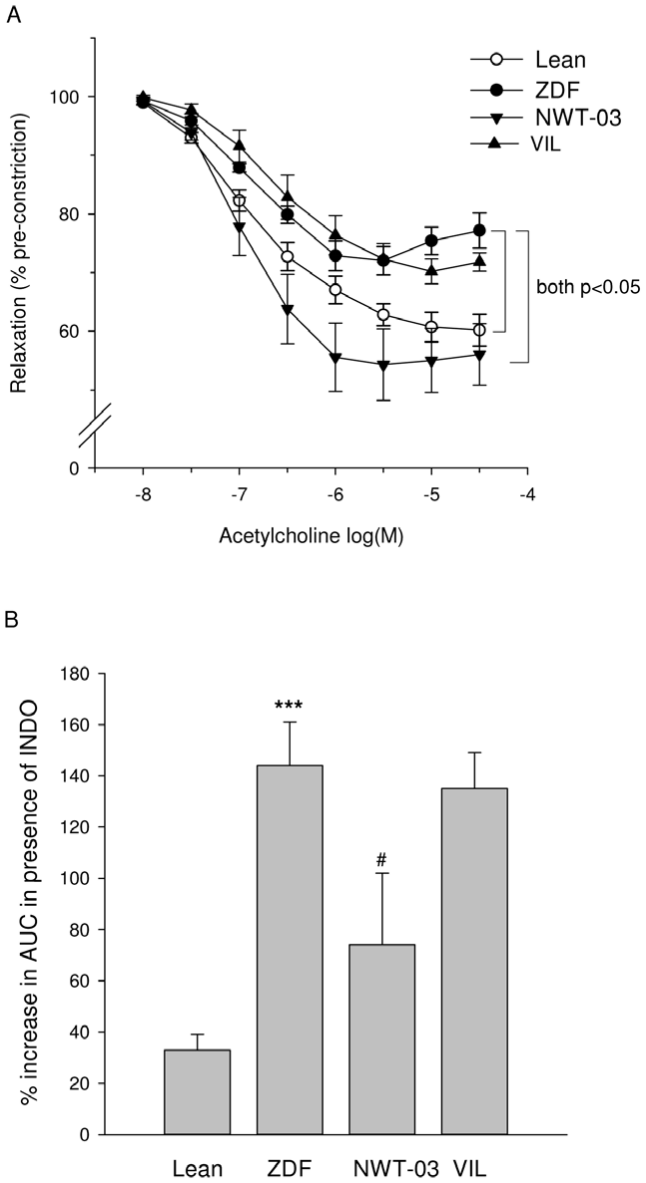
EDR response to ACh in isolated aortic rings. (A) ACh-induced relaxation in untreated ZDF rats was significantly reduced compared to that in lean controls. Relaxations were significantly increased in ZDF rats supplemented with NWT-03, but not in those treated with VIL. Pre-incubation with the COX-inhibitor indomethacin (INDO) profoundly improved relaxation to ACh in untreated ZDF rats (not shown in panel A; see lean vs. ZDF in panel B). The area under the curve (AUC) for ACh-induced relaxation in the absence and presence of INDO was determined for each group and used to calculate the percentage improvement. Data are means ± SEMs; significant differences are as indicated (repeated measures ANOVA). (B) Acute COX inhibition improved ACh-induced relaxation to a significantly smaller extent in ZDF rats supplemented with NWT-03, indicating a diminished suppression of relaxation by contractile PGs in the latter rats. Data are means ± SEMs (***p<0.001 vs. lean; #p<0.05 vs. ZDF).

EDR responses to ACh in aortas of ZDF rats were significantly impaired compared to those of lean rats (Figure 5A). Since maximal endothelium-independent relaxation to SNP was not affected (data not shown), this suggests an impairment at the level of the endothelium rather than a vascular smooth muscle cell defect. In the presence of indomethacin, a COX-inhibitor, the EDR response to ACh markedly improved in ZDF rats, indicating that contractile prostaglandins (PGs) opposed normal relaxation (Figure 5B).

### Effects of VIL and NWT-03 supplementation in ZDF rats (Table 2)

**Table 2:** Characteristics of 25-week-old ZDF rats after 15 weeks of treatment.

	ZDF (n = 7)	ZDF+NWT-03 (n = 9)	ZDF+VIL (n = 7)	One-way p-value
Body weight (g)	427±20	435±15	426±7	0.894
Left kidney weight (mg)	2154±47	2285±82	2274±56	0.387
Right kidney weight (mg)	2199±74	2369±68	2258±74	0.243
***Hemodynamic parameters***				
Aorta HR (bpm)	226±14	229±10	240±11	0.693
Aorta SBP (mmHg)	124±4	116±6	116±7	0.544
***Metabolic parameters***				
Glucose (mmol/L)	23.9±1.72	23.2±0.95	21.5±2.38	0.594
Hb1Ac (%)	9.54±0.16	9.33±0.20	9.00±0.20	0.189
Insulin (ng/mL)	28.5±4.6	38.4±5.2	33.6±4.4	0.374
Cholesterol (mmol/L)	7.33±0.19	7.05±0.31	6.52±0.49	0.312
FFAs (mmol/L)	0.66±0.14	0.89±0.26	0.30±0.12	0.231
GLP-1 (pmol/L)	6.22±0.38	8.66±1.49	32.0±6.0***	<0.001
***Urinary parameters***				
Albumin (mg/24 h)	77.4±15.7	43.9±4.5*	125.3±16.3*	<0.001
Glucose (category)	>7.0	>7.0	>7.0	n.d.
MDA:Creatinine	4.44±1.03	2.24±0.15*	3.52±0.44	0.035
24-h Urine volume (mL)	75±8	54±6	122±14**	0.001

Abbreviations: HR, heart rate; bpm, beats per minute; SBP, systolic blood pressure; FFAs, free fatty acids; GLP-1, glucagon-like peptide 1; MDA, malondialdehyde; n.d. not determined.

Data are means ± SEMs (*p<0.05, **p<0.01 and ***p<0.001 vs. untreated). P-values for one-way ANOVAs are indicated in the last column.

GLP-1 levels trended higher (∼1.4-fold) after NWT-03 treatment (although this difference did not reach statistical significance), and were profoundly increased (∼5-fold, p<0.001) by VIL. Despite these effects, neither agent improved any of the metabolic parameters studied. Although the diabetic state was not prevented, urinary MDA, an index of lipid peroxidation, was slightly lower after supplementation, particularly with NWT-03. Both VIL- and NWT-03-treated rats also showed significant improvement in certain parameters of renal inflammation and structural damage. Specifically, renal expression of IL-1β and IL-13 mRNA, representative indicators of inflammatory cytokine production, was reduced after VIL and NWT-03 (Figure 2), as was the severity of FGS (Figure 3). In conjuction herewith the expression of α-SMA and KIM-1 as markers of pre-fibrosis and tubular damage were also reduced, albeit of borderline significance. Interestingly, renal mRNA expression of the pro-inflammatory marker TNFα was also reduced, but only after NWT-03 treatment (Figure 2). The NWT-03-treated group was additionally distinguished by a reduction in renal P22*^phox^* and COX-1/2 protein expression, an action not produced by supplementation with VIL (Figure 4). Similarly, only NWT-03 reversed the decrease in renal TXA_2_R protein.

Aortic EDR responses to ACh after VIL were comparable to those in untreated ZDF rats, and were similarly sensitive to COX-inhibition with indomethacin. In contrast, EDR responses were improved after NWT-03 and were significantly less influenced by the presence of indomethacin (Figure 5).

## Discussion

In the present study, daily oral intake of NWT-03 for 15 weeks decreased renal damage in diabetic ZDF rats, reducing FGS and albuminuria by about 50%. In addition, impaired EDR responses to ACh, an indicator of endothelial dysfunction, was improved in ZDF rats after NWT-03. These findings demonstrate the beneficial effects of long-term NWT-03 supplementation in mitigating the development of renovascular damage in this preclinical model of DM2.

### Development of renovascular damage in the ZDF rat model of DM2

The present findings with ZDF rats are consistent with those reported previously. Already at a young age, obese ZDF rats showed profound hyperinsulinemia and the first signs of hyperglycemia, hyperglycation, and dyslipidemia. At the end of the study period, when rats were 25 weeks of age, serum insulin had declined to lean levels and blood glucose had dramatically increased. A similar relationship between insulinopenia due to exhaustion of pancreatic beta cell function and progressive, severe hyperglycemia has been observed previously, both in ZDF rats [17] and patients with DM2 [18]. Because of this physiological and metabolic profile, the ZDF rat is regarded a relevant model for humans with DM2 [19].

In addition to a metabolic DM2 profile, ZDF rats develop progressive nephropathy and renal failure [17], [20]–[22]. Albuminuria, the earliest detectable clinical abnormality in diabetic glomerulopathy, increased over time and was accompanied by a ∼5-fold increase in FGS at the end of the study period. At that time, urinary MDA was also increased in ZDF rats, as was renal abundance of the P22*^phox^* subunit of NAD(P)H oxidase. This is in line with previous reports depicting the ZDF rat as a model of increased oxidative stress [22], [23]. Enhanced production of reactive oxygen species (ROS) and advanced glycation end products induced by high glucose are believed to elicit inflammation and alter gene expression of growth factors and cytokines implicated in diabetic nephropathy. Consistent with this, renal mRNA analyses suggested an increase in the expression of VCAM-1 with concomitant infiltration of white blood cells (CD68) and production of inflammatory cytokines (TNFα, Il-1β, Il-13) in ZDF rats, resulting in injury to renal cells and a phenotypic change in matrix-forming myofibroblasts (staining for KIM-1 and αSMA).

COX-derived PGs play important roles in maintaining renal function, but may also be involved in the pathogenesis of diabetic nephropathy [24]–[26]. Komers et al. [27] reported an increase in kidney abundance of COX-2 protein in 4- and 12-week-old ZDF rats that was associated with enhanced urinary excretion of PGs and parallel metabolic abnormalities. In the present study, the renal abundance of both COX-1 and -2 protein were increased in 25-week-old ZDF rats, whereas TXA_2_R protein expression was decreased. This latter effect (PG receptor down-regulation) may reflect a response to increased production of PGs following up-regulation of COX protein. Notably, the impaired EDR response to ACh in the present study was fully restored in ZDF aortic rings pre-incubated with indomethacin, a non-selective inhibitor of COX. Recently, Retailleau et al. [28] reported elevated plasma TXB_2_ (a TXA_2_ metabolite) in association with increased vascular superoxide production and COX-2 expression in mesenteric arteries of ZDF rats, and reduced AT_2_ receptor-mediated vasorelaxation. Dilation in that study returned to the control level after acute superoxide reduction, COX-2 inhibition, or TXA_2_ synthesis inhibition. Although we did not analyze aortic protein abundance, the similar directionalities of renal COX protein expression and functional effects of vascular COX-inhibition in the present study appear to mutually reinforce the role of COX-derived PGs in the development of renovascular damage in this model.

### Long-term effects of VIL and NWT-03

The starting point for this study was the previous demonstration that NWT-03 exhibited mild *in vitro* and *in vivo* DPP4- and ACE-inhibitory activities, both of which could, in theory, help to improve glycemic control and/or limit renovascular end-organ damage in DM2. The effects of ACE-inhibitor therapy in ZDF rats have been studied to a certain extent, but relatively few studies have addressed the effects of chronic DPP4-inhibitor treatment on renovascular damage in this model. Therefore, as a frame of reference for the effects of supplementation with NWT-03 on the development renovascular damage in the present study, a parallel group of ZDF was treated with the DPP4-inhibitor VIL.

DPP4-inhibitors—the latest addition to the list of available antidiabetes agents—increase the activity of GLP-1 and glucose-dependent insulinotropic peptide and have been found effective in lowering glycated hemoglobin, and fasting and postprandial glucose levels [29], [30]. In insulin-resistant, non-diabetic Zucker fatty rats, VIL, delivered as a single oral dose, was shown to induce a dose-dependent inhibition of DPP4, enhance glucose-induced increases in intact GLP-1, decrease postload glucose excursions, and increase the insulinogenic index; the maximally effective dose was reported to be 3 mg/kg, and nearly identical effects were obtained with chronic treatment [8]. In the present study with diabetic ZDF rats, chronic administration of VIL at the same dose profoundly increased basal GLP-1 levels. But notwithstanding this action, fasting blood glucose and HbA1c were not significantly changed. In a previous study in ZDF rats, chronic treatment with a different DPP4-inhibitor (FE 999011; 10 mg/kg per os, twice a day) delayed, but did not prevent, the onset of hyperglycemia [31]. Our results may also be in line with a recent study by Thomas et al. [32] in which chronic treatment with VIL (3 mg/kg/day for 5 weeks) reduced postload glucose excursion in ZDF rats, but not the 24-hour glucose profile in the fed state or HbA1c levels. In that study, only chronic treatment with the long-acting DPP4 inhibitor BI 1356 resulted in an overall glucose-lowering effect, whereas the short-acting VIL did not.

Interestingly, FGS and renal tissue Il-1β and Il-13 mRNA expression were reduced after VIL in the present study. This suggests an action of VIL independent of its modulation of metabolic parameters. DPP4 is abundantly expressed in the proximal tubules in the kidney; and in human glomerular endothelial cells, high glucose may increase the expression and activity of DPP4 [33]. Furthermore, the CD26/DPP4 system is known act through its DPP4 enzymatic activity to play a key role in immune function as a T-cell activating molecule and regulator of the functional effects of selected biological factors [34]. To our knowledge, this is the first study reporting attenuated FGS together with reduced renal Il-1β and Il-13 mRNA expression after chronic treatment with the DPP4 inhibitor VIL in ZDF rats. Of note, NWT-03 exerted the same effects, yet without significantly altering circulating GLP-1 levels. Taken together, these findings suggest that VIL and NWT-03 may share a similar mode of action on the kidney that is independent of systemic DPP4 inhibition and/or metabolic improvement. One plausible explanation is that these agents acted locally in renal tissue, a possibility that may be the subject of future studies. Such studies may additionally include comparisons to incretin mimetics such as exenetide to further mechanistically detail the effects of DPP4-inhibition versus per se GLP-1 receptor agonism in diabetic kidney.

Unlike VIL, NWT-03 also normalized renal TNFα mRNA and P22*^phox^* protein expression, and significantly reduced urinary MDA. Furthermore, increased renal COX-1/2 protein abundance was normalized, and urinary albumin excretion was reduced after NWT-03, but not VIL. Hence, it seems unlikely that these latter effects of NWT-03 resulted from (local) DPP4 inhibition. Instead, it is more likely that they are related to the ACE-inhibitory activity of NWT-03 we observed in preliminary studies. Angiotensin II—acting via increased production of autocrine factors, such as TNFα, and up-regulation of NAD(P)H oxidase subunits and production of ROS—has a key role in renal injury and in the progression of chronic renal diseases of diverse etiologies [35]. Angiotensin II-mediated inflammation and oxidative stress may contribute to up-regulation of COX-2, whose expression is induced by ROS [36], [37]. Conversely, blocking the AT1 receptor blocker has been shown to reverse up-regulation of renal COX-2 and other pro-oxidant/inflammatory systems in obese Zucker rats, and prevent the associated glomerulopathy and proteinuria [38].

The enhancing effect of NWT-03 on endothelial function observed here is also more likely attributable to the ACE-inhibitory properties of NWT-03 than inhibition of DPP4, given the lack of an effect of VIL on EDR. ACE inhibitors and AT1 receptor blockers prevent hyperglycemia-induced oxidative stress by modulating angiotensin action and production; this is of particular interest because hyperglycemia is able to directly modulate cellular angiotensin generation [39]. In a study by Oltman et al. [40] in ZDF rats, treatment with the ACE inhibitor enalapril reduced vascular oxidative stress and improved mesenteric and coronary endothelial dysfunction independent of changes in metabolic parameters (blood glucose, cholesterol, triglycerides, and FFAs), which were not significantly reduced by ACE inhibition. Notably, not only was the EDR response to ACh significantly enhanced after NWT-03 in the current study, its acute modulation by indomethacin was markedly smaller, suggesting that functional down-regulation of COX-derived contractile PGs by NWT-03 contributed to improved vascular endothelial function. This effect of NWT-03 occurred in parallel with reduced renal COX protein expression, and both actions might be accounted for by inhibition of ACE (as suggested above). Whether NWT-03 also exerts direct COX-inhibitory effects and/or whether other additional mechanisms may contribute to its long-term effects should be addressed in future studies.

In summary, dyslipidemia and prolonged hyperglycemia promote an increase in oxidative stress, inflammation and vascular damage, which together underlie diabetic nephropathy and promote endothelial dysfunction and the associated micro- and macrovascular complications. Employing the ZDF rat as a preclinical model of DM2, we found that (1) long-term supplementation with NWT-03 exerted a beneficial effect by attenuating renal damage development and preventing aortic endothelial dysfunction, likely through a mechanism involving decreased oxidative stress and down-regulation of COX expression, and (2) VIL produced a favorable effect—albeit it more limited—on renal pro-inflammatory cytokines and glomerulosclerosis independent of systemic improvement of metabolic parameters. Based on a comparison to the DPP4-inhibitor VIL, the results suggest that the effects of NWT-03 in the present study were related to its ACE-inhibitory activity in addition to its DPP4-inhibitory properties. As advanced by the American Diabetes Society [41], medical nutrition therapy is important in preventing diabetes, managing existing diabetes, and preventing, or at least slowing, the rate of development of diabetes complications. This therapeutic strategy includes secondary and tertiary interventions through healthy nutrition and functional foods for individuals with diabetes and seeks to prevent (secondary) or control (tertiary) complications of diabetes. By analogy with current trends in the field of drug research [9], the development and analysis of protein hydrolysates that follow a multiple-targeting strategy may prove to be of benefit in functional food formulations.

## Supporting Information

Figure S1 **Acute effects of oral NWT-03 and VIL on plasma DPP4 activity and blood pressure in ZDF rats.** DPP4 activity and blood pressure were determined 1.5 hours after administering NWT-03 (1 g/kg) or VIL (3 mg/kg) in 1 mL water to anesthetized ZDF rats via a gastric tube. (A) Plasma DPP4-activity was mildly inhibited in ZDF rats receiving NWT-03 (∼25% decrease from baseline) and was profoundly inhibited in those receiving VIL (∼65% decrease from baseline), but was unaffected in those receiving water only. (B) Systolic blood pressure was unaffected by administration of NWT-03 or VIL. Data are means ± SEMs (*p<0.05 for ZDF vs. ZDF+NWT-03; ***p<0.001 for ZDF vs. ZDF+VIL; n = 6–9 per group).
(TIF)

Figure S2 **Acute effect of oral NWT-03 on plasma ACE activity and blood pressure in SHRs.** ACE activity and blood pressure were determined 1.5 hours after administering NWT-03 (1 g/kg) in 1 mL water to anesthetized SHRs via a gastric tube. (A) Plasma ACE activity was mildly inhibited (∼25% decrease from baseline) and (B) systolic blood pressure was reduced (∼30% decrease from baseline) in animals receiving NWT-03, but not in those receiving water only. Data are means ± SEMs (*p<0.05 for SHR vs. SHR+NWT-03; n = 5 per group).
(TIF)

Table S1 **Primers used for real-time PCR.**
(DOC)
