# Supplementary figures and images for: Attenuation of Renovascular Damage in Zucker Diabetic Fatty Rat by NWT-03, an Egg Protein Hydrolysate with ACE- and DPP4-Inhibitory Activity
Source: PLoS One. 2012 Oct 10;7(10):e46781. doi: 10.1371/journal.pone.0046781 (PMC3468629; doi:10.1371/journal.pone.0046781)

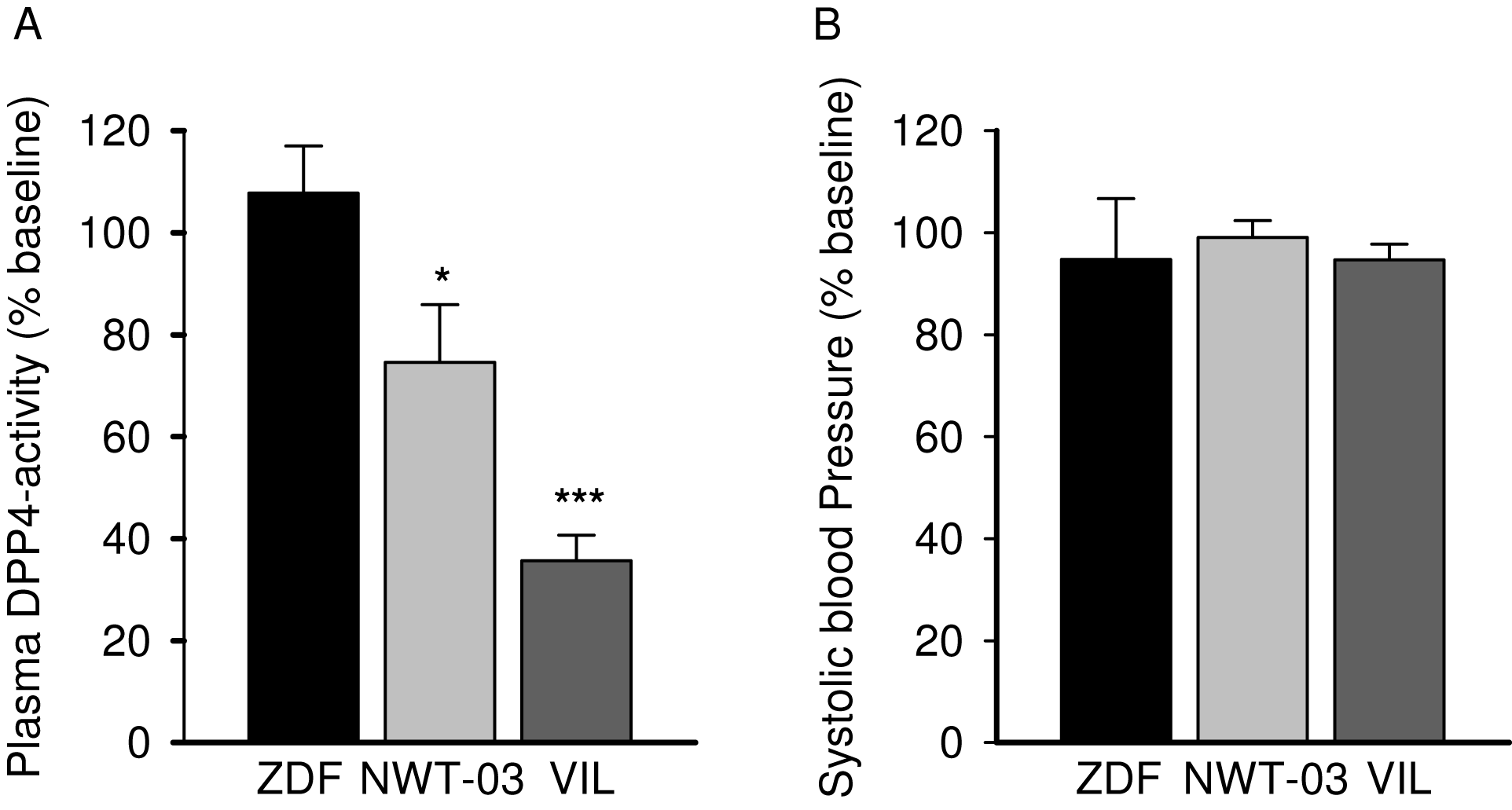

Supplement: Figure S1 — Acute effects of oral NWT-03 and VIL on plasma DPP4 activity and blood pressure in ZDF rats. DPP4 activity and blood pressure were determined 1.5 hours after administering NWT-03 (1 g/kg) or VIL (3 mg/kg) in 1 mL water to anesthetized ZDF rats via a gastric tube. (A) Plasma DPP4-activity was mildly inhibited in ZDF rats receiving NWT-03 (∼25% decrease from baseline) and was profoundly inhibited in those receiving VIL (∼65% decrease from baseline), but was unaffected in those receiving water only. (B) Systolic blood pressure was unaffected by administration of NWT-03 or VIL. Data are means ± SEMs (*p<0.05 for ZDF vs. ZDF+NWT-03; ***p<0.001 for ZDF vs. ZDF+VIL; n = 6–9 per group). (TIF) [file pone.0046781.s001.tif]

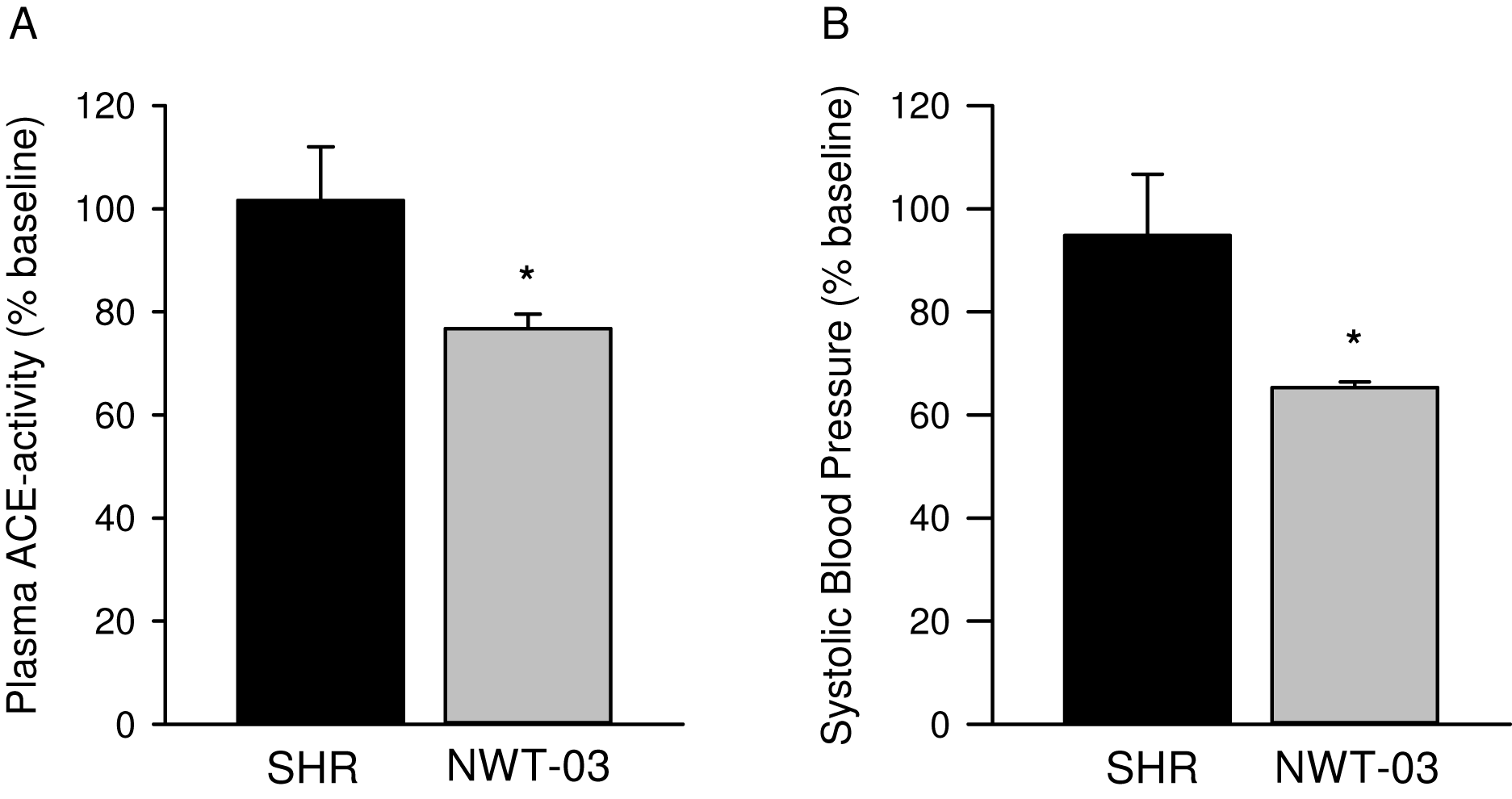

Supplement: Figure S2 — Acute effect of oral NWT-03 on plasma ACE activity and blood pressure in SHRs. ACE activity and blood pressure were determined 1.5 hours after administering NWT-03 (1 g/kg) in 1 mL water to anesthetized SHRs via a gastric tube. (A) Plasma ACE activity was mildly inhibited (∼25% decrease from baseline) and (B) systolic blood pressure was reduced (∼30% decrease from baseline) in animals receiving NWT-03, but not in those receiving water only. Data are means ± SEMs (*p<0.05 for SHR vs. SHR+NWT-03; n = 5 per group). (TIF) [file pone.0046781.s002.tif]
