# Supplementary material for: Attenuation of Renovascular Damage in Zucker Diabetic Fatty Rat by NWT-03, an Egg Protein Hydrolysate with ACE- and DPP4-Inhibitory Activity
Source: PLoS One. 2012 Oct 10;7(10):e46781. doi: 10.1371/journal.pone.0046781 (PMC3468629; doi:10.1371/journal.pone.0046781)
Supplement: Table S1 — Primers used for real-time PCR. (DOC) [file pone.0046781.s003.doc]

Table S1. Overview of the primers used for real time PCR

| Gene | Primer | |
| --- | --- | --- |
|  | forward: 5'→ 3' | reverse: 5'→ 3' |
| Il-1β | CTGTGGCAGCTACCTATGTC | CACACTAGCAGGTCGTCATC |
| Il-13 | CTTGCCAACACTGTCTACAC | CGGCTCCTTACCTATACTCA |
| E-selectin | CCATTCGGCCTCTTCAGCTA | TGCAGCTCACAGAGCCATTC |
| VCAM-1 | TAAGTTACACAGCAGTCAAATG | CACATACATAAATGCCGGAATC |
| CD68 | AAGCAGCACAGTGGACATTC | ATGATGAGAGGCAGCAAGAG |
| TNFα | GTACCACCAGTTGGTTGTCT | CACGCTCTTCTGTCTACTGA |
